# Quantum and nanoscale sensing of microinflammation for earlier detection of chronic inflammatory and neuroimmune disease

**DOI:** 10.1186/s11671-026-04830-0

**Published:** 2026-08-11

**Authors:** Yuki Tanaka, Yaze Wang, Hiroki Tanaka, Shintaro Hojyo, Naoko Kamiya, Kaoru Murakami, Masaaki Murakami

**Affiliations:** 1 Quantum Immunology Team, Institute for Quantum Life Science, National Institutes for Quantum Science and Technology (QST), Chiba, Japan; 2 Division of Molecular Psychoneuroimmunology, Institute for Genetic Medicine and Graduate School of Medicine, Hokkaido University, Kita‑15, Nishi‑7, Kita‑Ku, Sapporo, 060‑0815 Japan; 3 Division of Microbial Oncology, Institute for Genetic Medicine, Hokkaido University, Sapporo, Hokkaido Japan; 4 Center of Infection-Associated Cancer, Institute for Genetic Medicine, Hokkaido University, Kita-ku, Sapporo, Japan; 5 Department of Microbiology, Graduate School of Medicine, The University of Tokyo, Bunkyo-ku, Tokyo, Japan; 6 Division of Molecular Neuroimmunology, National Institute for Physiological Sciences, National Institutes of Natural Sciences, Okazaki, Japan; 7 Institute for Vaccine Research and Development (HU‑IVReD), Hokkaido University, Sapporo, Japan

## Abstract

Chronic inflammatory diseases may begin with spatially restricted and low-abundance inflammatory events that precede overt symptoms. In this perspective, we refer to such early events as micro-inflammation and discuss how mechanistic insights from the IL-6 amplifier and neuroimmune regulation may guide earlier detection of related diseases. The IL-6 amplifier is a positive-feedback inflammatory circuit in non-immune cells driven by concurrent NF-κB and IL-6–STAT3 signaling, whereas the gateway reflex illustrates how defined neural inputs can induce site-specific vascular micro-inflammation and immune-cell entry into affected tissues and organs. We also discuss stress-associated neuroimmune remodeling in diffuse neuropsychiatric systemic lupus erythematosus (dNPSLE) as a representative example of region-specific brain inflammation that may precede overt neuropsychiatric manifestations. On this basis, we examine how quantum and nanoscale sensing platforms, particularly fluorescent nanodiamonds containing nitrogen-vacancy centers and AI-assisted nanopore devices, could be adapted to detect candidate readouts of micro-inflammation, including reactive oxygen species, cytokine-related signals, nucleic acids, and other trace biomarkers. The original contribution of this Perspective is a mechanistically guided framework that maps micro-inflammatory biology related to the IL-6 amplifier, the gateway reflex, and dNPSLE onto specific classes of candidate nanosensing readouts, including oxidative stress-related physicochemical signals, cytokine-associated soluble biomarkers, extracellular nucleic acids, and other trace molecular signatures. Rather than providing a comprehensive review of all inflammatory mechanisms or diagnostic technologies, we present a mechanistically focused perspective linking neuroimmunology to next-generation sensing. We also highlight current limitations, including the predominantly preclinical nature of supporting evidence, uncertainty regarding accessible blood or cerebrospinal fluid biomarkers at presymptomatic stages, and the challenge of translating spatially restricted inflammatory signals into non-invasive human diagnostics.

## Introduction

Chronic inflammation contributes not only to autoimmune diseases such as rheumatoid arthritis (RA), but also to the onset and progression of a wide range of disorders, including neurodegenerative, neuropsychiatric, and metabolic diseases [1]. A major challenge for early diagnosis is that disease-associated inflammatory events often arise as spatially restricted, low-abundance, and transient changes before overt clinical manifestations. In this article, we refer to such early localized events as micro-inflammation, thereby distinguishing them from generalized systemic low-grade inflammation. From the standpoint of nano-enabled diagnostics, these features make chronic inflammation a particularly difficult target, because conventional assays and imaging methods often fail to detect inflammatory processes until they have progressed to tissue-damaging states. Although chronic inflammation has traditionally been attributed mainly to dysregulated immune-cell networks initiated by T cells, including autoreactive populations [2, 3], increasing evidence indicates that its persistence and tissue specificity are critically shaped by non-immune cells, including tissue-resident cells, vascular endothelial cells, and fibroblasts [4–6]. One mechanistic framework for this concept is the IL-6 amplifier, a positive-feedback inflammatory circuit in non-immune cells driven by the simultaneous activation of NF-κB and IL-6–STAT3 signaling [7–9]. Activation of this pathway promotes sustained production of cytokines and chemokines, thereby converting subtle local perturbations into self-reinforcing inflammatory niches that recruit immune cells and amplify tissue inflammation. In this sense, the IL-6 amplifier provides a useful model for understanding how micro-inflammatory seeds may arise and persist before overt chronic inflammatory disease becomes clinically apparent. At the same time, the molecular detection of these early events remains technically challenging, and methods capable of sensitively capturing IL-6 amplifier-associated changes at presymptomatic stages are still limited.

The central nervous system (CNS) offers a particularly informative setting in which to study the spatial organization of micro-inflammation. Under physiological conditions, the blood-brain barrier (BBB), which is formed by endothelial cells together with supporting cellular and matrix components, tightly regulates the trafficking of immune cells and macromolecules and thereby preserves CNS homeostasis [10, 11]. Nevertheless, under defined pathological conditions, blood-derived immune cells can enter the CNS and contribute either to host defense or to neuroinflammatory and autoimmune disease.

Multiple sclerosis (MS) is a prototypical autoimmune disease of the CNS in which autoreactive CD4 + T cells recognizing myelin antigens infiltrate the CNS and induce demyelination and neurological dysfunction [12, 13]. Studies using experimental autoimmune encephalomyelitis (EAE) led to the identification of the gateway reflex, a neuroimmune mechanism in which specific neural inputs induce discrete vascular “gates” that permit selective entry of blood immune cells, including autoreactive T cells, into the CNS. Subsequent work has shown that diverse stimuli, including gravity, electrical stimulation, pain, stress, light exposure, and joint inflammation, can induce gate formation at distinct vascular sites [14–18]. These findings suggest that micro-inflammation is not only molecularly amplified but also spatially regulated by neural activity. However, most of the supporting evidence for these mechanisms has been obtained in animal models, and the extent to which analogous site-specific vascular inflammatory programs can be detected or monitored in humans remains an important translational question.

Neural regulation of micro-inflammation is not limited to vascular entry sites but may also involve broader remodeling of inflammatory circuits within the CNS. In this context, diffuse neuropsychiatric systemic lupus erythematosus (dNPSLE) provides a representative example in which stress-associated neuroimmune remodeling has been linked to pathogenic brain inflammation [19]. Experimental studies suggest that excessive stress can promote dNPSLE-like pathology through IL-12/23-associated neuroimmune circuit remodeling, supporting the idea that localized inflammatory programs may precede overt neuropsychiatric manifestations. At the same time, dNPSLE is biologically heterogeneous, and this pathway should be regarded as one candidate mechanism within a broader inflammatory landscape. Additional processes, including altered microglial states, peripheral immune-cell infiltration, and other cytokine- or complement-associated pathways, may also contribute to dNPSLE pathogenesis.

These biological insights motivate the search for measurable features of micro-inflammation that could support earlier diagnosis. Potential readouts may include oxidative stress, cytokine-associated molecular signatures, extracellular nucleic acids, and other trace biomarkers in affected tissues, blood, or cerebrospinal fluid. A key challenge is that many of these signals are expected to be transient, low in abundance, and spatially restricted, raising uncertainty about which biomarkers are accessible in presymptomatic human disease and in which biological compartments.

Accordingly, the original contribution of this Perspective is a mechanistically guided framework that maps micro-inflammatory biology related to the IL-6 amplifier, the gateway reflex, and dNPSLE onto specific classes of candidate nanosensing readouts, including fluorescent nanodiamond (FND)-accessible physicochemical signals and nanopore-accessible extracellular nucleic-acid or affinity-based biomarker signals. We therefore present a focused perspective on how these mechanisms can inform next-generation sensing strategies for chronic inflammatory and neuroimmune disease, while also discussing the biological and translational limitations that must be addressed before such approaches can be applied to human diagnostic use.

## The IL-6 amplifier as a mechanistic framework for chronic inflammation

Chronic inflammation is a pathogenic state implicated in a wide range of disorders, including autoimmune diseases such as RA, metabolic diseases such as metabolic syndrome, neurodegenerative diseases exemplified by Alzheimer’s disease, and neuropsychiatric disorders including schizophrenia and depression [8, 20–23]. In the treatment of RA, conventional immunosuppressive agents have long been used as standard therapy, but biologic agents have substantially changed the therapeutic landscape. In particular, the clinical efficacy of the anti–IL-6 receptor antibody tocilizumab suggests that persistent IL-6-dependent signaling is not merely a downstream consequence of inflammation, but it can also contribute to disease progression.

IL-6 signaling occurs through at least two related modes [9, 24–27]. In classic signaling, IL-6 binds to the membrane-bound IL-6 receptor (IL-6R), and the resulting complex associates with gp130 to activate intracellular pathways centered on JAK/STAT3. In trans-signaling, IL-6 binds to soluble IL-6R, thereby enabling gp130-expressing cells that do not express membrane-bound IL-6R to respond to IL-6. This distinction is relevant to chronic inflammation because trans-signaling can broaden IL-6 responsiveness to non-immune cells, including endothelial cells and fibroblasts, whereas classic signaling may also contribute to cell-type-specific physiological or autocrine responses. Thus, the IL-6 amplifier should not be regarded as an alternative receptor-signaling mode, but rather as a localized feed-forward inflammatory circuit in which IL-6–STAT3 signaling, initiated through classic and/or trans-signaling depending on the tissue context, cooperates with NF-κB activation in non-immune cells to sustain chemokine production, immune-cell recruitment, and inflammatory persistence.

To investigate how sustained IL-6 signaling contributes to chronic inflammation, gp130 Y759F knock-in mice (F759 mice) were established as an IL-6-dependent arthritis model [28]. Tyrosine 759 of gp130 normally serves as a binding site for SOCS3 and mediates negative feedback regulation of IL-6 signaling. In F759 mice, substitution of this residue with phenylalanine disrupts that feedback and results in prolonged IL-6 signaling. These mice spontaneously develop IL-6-dependent, bilaterally symmetrical RA-like arthritis with age, providing a useful model for examining how persistent IL-6 activity in vivo promotes chronic inflammatory pathology [8].

Studies in this model showed that age-dependent activation of CD4 + T cells is closely linked to sustained IL-6–STAT3 signaling in non-immune cells rather than immune cells alone. Bone marrow transplantation experiments indicated that IL-6–STAT3 activation in non-immune cells, including endothelial cells and fibroblasts, promotes IL-7 production, which in turn supports the expansion of activated CD4 + T cells [25]. Consistent with this interpretation, neutralization of IL-7 suppressed both CD4 + T-cell expansion and arthritis progression in F759 mice [29]. In addition, many of the activated CD4 + T cells that accumulated with age in this model displayed a T helper 17 (Th17) phenotype [29, 30].

These observations led to further examination of the interaction between IL-6 and IL-17 A, a cytokine produced by Th17 cells that activates NF-κB signaling [31, 32]. In fibroblasts, co-stimulation with IL-6 and IL-17 A induced markedly greater IL-6 production than either stimulus alone, indicating that simultaneous activation of STAT3 and NF-κB can generate a positive-feedback loop in non-immune cells [7–9]. This inflammation-amplifying mechanism was designated the “IL-6 amplifier.” In the present context, the IL-6 amplifier can be understood as a localized inflammatory circuit in which non-immune cells integrate cytokine and inflammatory inputs to sustain chemokine production, immune-cell recruitment, and tissue-specific inflammatory persistence.

**Fig. 1:**
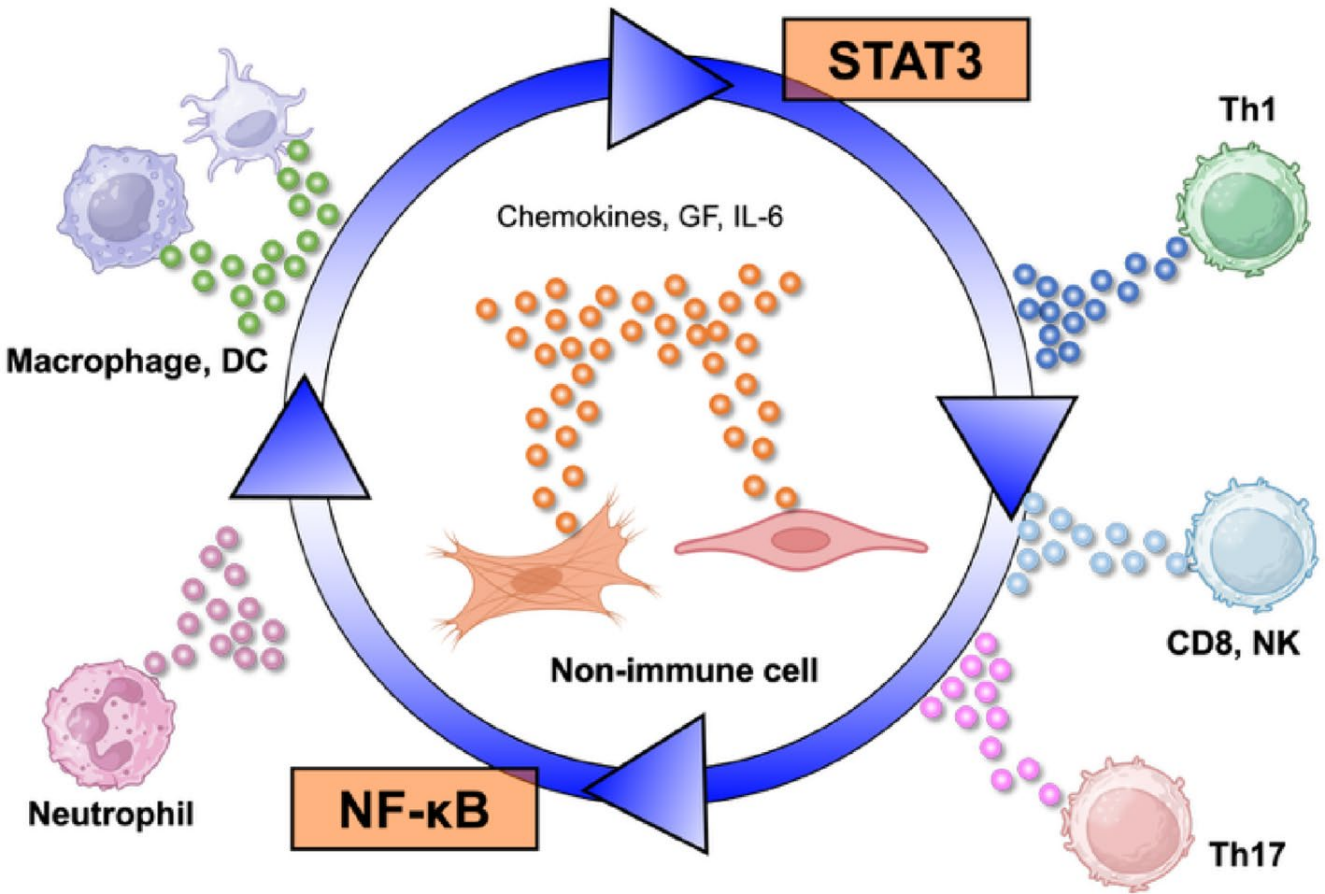
Conceptual framework of the IL-6 amplifier. Concurrent activation of NF-κB and STAT3 signaling pathways in non-immune cells leads to sustained production of inflammatory cytokines and chemokines. This cooperative signaling establishes a positive feedback loop that amplifies NF-κB activity, thereby creating a self-sustaining inflammatory microenvironment. Such IL-6 amplifier–driven responses in tissue-resident non-immune cells have been implicated as a common molecular basis underlying the initiation and progression of diverse chronic inflammatory diseases. CD8 + T cell, CD8 + cytotoxic T lymphocyte; DC, dendritic cell; NK, natural killer cell; Th, T helper cell

Subsequent studies suggest that this mechanism is not restricted to fibroblasts or endothelial cells but that it can also be activated in tissue-specific non-immune cells, including renal tubular epithelial cells, keratinocytes, airway basal cells, and salivary gland epithelial cells (Fig. 1). Genome-wide screening further identified a large number of positive regulators of IL-6 amplifier activity, many of which are linked to human disease [7]. Together, these findings support the view that the IL-6 amplifier represents a broadly relevant mechanistic framework for chronic inflammation.

Micro-inflammation associated with the IL-6 amplifier refers to spatially restricted, early inflammatory states characterized by local co-activation of NF-κB and STAT3, induction of IL-6 and chemokines, activation of stromal or endothelial cells, and recruitment of immune cells before overt tissue damage or systemic biomarker elevation becomes evident. These features make IL-6 amplifier-associated molecules attractive candidates for earlier detection of disease. However, most direct evidence for amplifier-associated states derives from experimental systems. Whether equivalent signatures can be robustly detected in presymptomatic human blood, cerebrospinal fluid, or other accessible samples remains uncertain and requires further investigation.

## The gravity-dependent gateway reflex

A central question in MS is how pathogenic immune cells gain access to the CNS across the BBB. Although myelin antigens are widely distributed throughout CNS white matter, inflammatory lesion localization is heterogeneous among patients [13, 33, 34]. This discrepancy suggests that blood immune-cell entry into the CNS is not spatially uniform but is instead regulated at specific vascular sites. This matter is also clinically relevant because currently available therapies that restrict immune-cell trafficking into the CNS, such as fingolimod and natalizumab, are effective but can be associated with systemic immunosuppression and serious adverse effects [35–38]. These considerations raise the possibility that more localized and site-specific regulation of immune-cell entry may exist.

Using an adoptive-transfer experimental autoimmune encephalomyelitis (EAE) model, in which myelin-reactive pathogenic CD4 + T cells are intravenously transferred, preclinical whole-mount immunohistochemical analyses revealed that these cells preferentially accumulated at the dorsal blood vessels of the fifth lumbar (L5) spinal cord rather than in the brain or upper spinal cord [14]. At these L5 dorsal vessels, expression of multiple chemokines, including CCL20, which selectively attracts Th17 cells, was markedly elevated [14]. Consistently, administration of neutralizing anti-CCL20 antibodies or the use of CCR6-deficient CD4 + T cells significantly reduced pathogenic T-cell accumulation at this site [14]. Notably, even in the absence of EAE induction, basal chemokine expression was higher at the L5 dorsal vessels than at upper spinal levels such as L1 [14], suggesting that this vascular region possesses a distinct predisposition to inflammatory activation. These observations support the idea that site-specific vascular micro-inflammation can function as an early disease-relevant “seed” before overt neurological symptoms appear. The robust chemokine production observed at the L5 dorsal vessels is therefore thought to reflect localized activation of the IL-6 amplifier.

The gravity-dependent gateway reflex was originally defined on the basis of this L5 vascular phenomenon, in which regional neural activation establishes a disease-relevant gateway for autoreactive T-cell entry across the BBB [14, 39, 40]. Anatomically, sensory neurons in the L5 dorsal root ganglion (DRG) innervate the soleus muscles, which function as major anti-gravity muscles and remain tonically active under normal gravitational loading [14, 41]. On the basis of this relationship, gravity-dependent sensory input was hypothesized to regulate local inflammation at the L5 dorsal vessels. Consistent with this idea, hindlimb unloading by tail suspension markedly reduced chemokine expression and pathogenic CD4 + T-cell accumulation at the L5 dorsal vessels and also decreased expression of the neuronal activation marker c-Fos in the L5 DRG [14]. Conversely, weak electrical stimulation of the soleus muscles in tail-suspended mice restored chemokine production, pathogenic T-cell accumulation, and c-Fos expression at the L5 dorsal vessels [14].

**Fig. 2:**
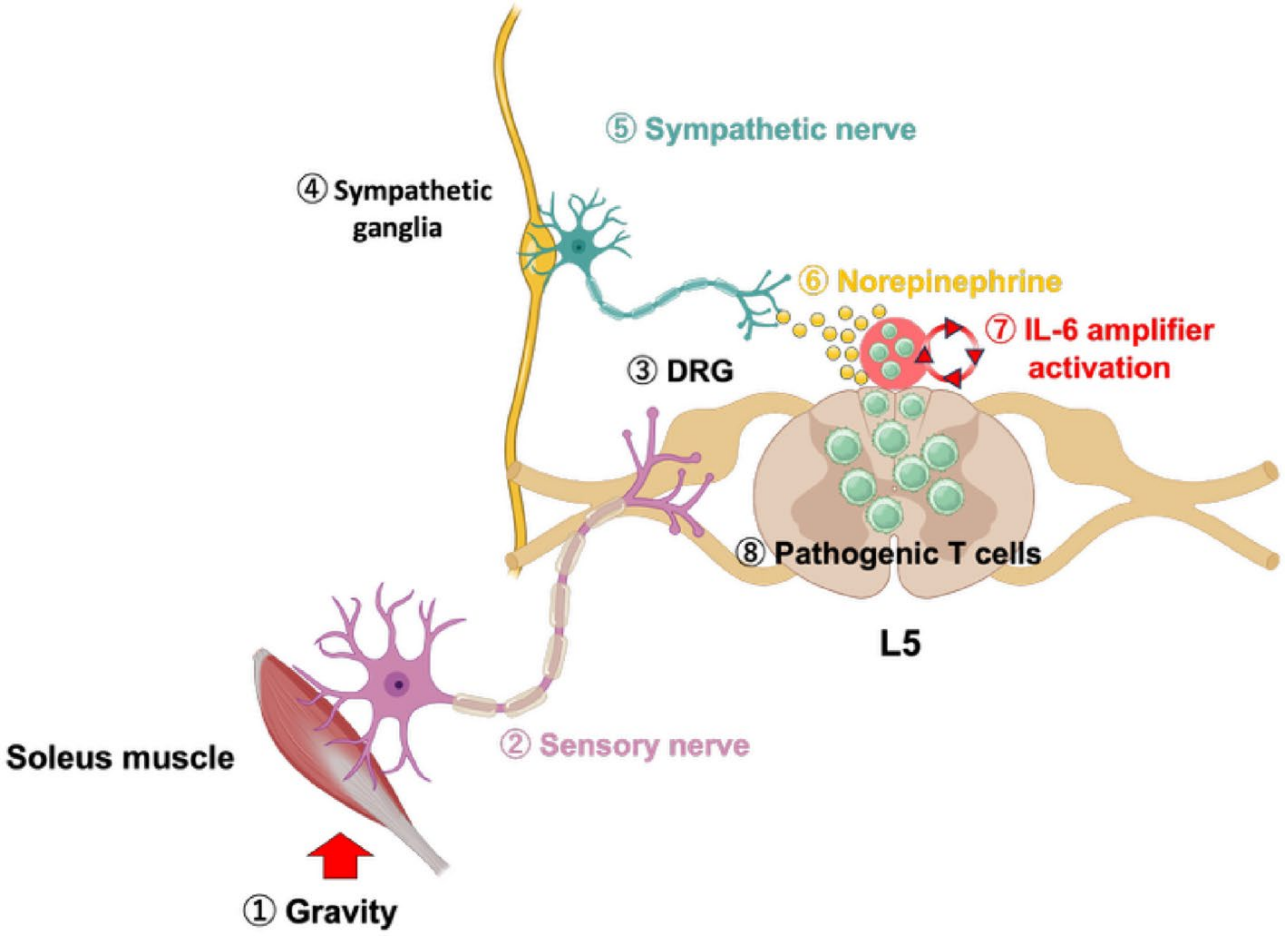
Gravity-dependent gateway reflex as a neuroimmune regulatory mechanism. Gravity stimulation activates sensory neurons innervating the soleus muscles, which in turn engage sympathetic neural pathways projecting to dorsal blood vessels at the L5 spinal level. Sympathetic neurotransmission, including local release of norepinephrine, promotes activation of the IL-6 amplifier in L5-specific vascular regions, leading to localized chemokine production such as CCL20. This spatially restricted inflammatory milieu facilitates selective accumulation of pathogenic CD4⁺ T cells at the L5 spinal cord, illustrating how neural inputs define vascular “gateways” for immune cell entry into the central nervous system

Additional analyses implicated sympathetic pathways in this response. Neuronal activation was higher in sympathetic ganglia at the L5 level than at upper spinal segments, and chemical sympathectomy suppressed chemokine expression, pathogenic CD4 + T-cell accumulation at the L5 dorsal vessels, and EAE development [14]. Together, these findings indicate that the gravity-dependent gateway reflex is mediated by coordinated sensory and sympathetic neural circuits that induce IL-6 amplifier-associated chemokine expression at specific L5 dorsal vessels, thereby establishing a localized gateway for immune-cell entry into the CNS (Fig. 2). Subsequent studies on pain-, stress-, light-, and joint inflammation-associated gateway reflexes further suggest that this mechanism represents a broader neuroimmune principle in which distinct environmental inputs create or suppress immune-cell gateways at different barrier sites [15–18, 42, 43].

At present, however, direct non-invasive detection of focal inflammatory events at sites analogous to the L5 dorsal vessels is not established in humans. A more realistic near-term clinical strategy may therefore seek indirect surrogate readouts in accessible compartments such as blood, or, in the future, to visualize secondary barrier-associated changes by high-resolution imaging, rather than to directly measure the vascular niche itself. Cerebrospinal fluid may also provide mechanistic information, although acquiring such samples is invasive. Thus, the gravity-dependent gateway reflex serves as a mechanistic model of disease-seeding micro-inflammation and as a guide for biomarker discovery.

## Stress-associated neuroinflammation and dNPSLE-related mechanisms

**Fig. 3:**
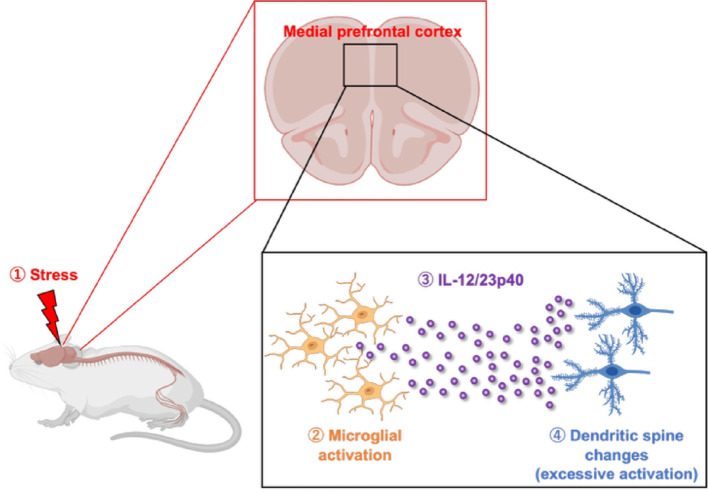
Stress-dependent neuroimmune remodeling in an experimental model of dNPSLE. MRL/lpr mice, which spontaneously develop systemic lupus erythematosus–like pathology, exhibit diffuse neuropsychiatric manifestations when subjected to chronic stress conditions. Under stress exposure, enhanced microglial activation and elevated expression of the inflammatory cytokine subunit IL-12/23 p40 are observed in specific brain regions. Pharmacological intervention targeting IL-12/23 signaling attenuates these neurobehavioral abnormalities and associated dendritic spine changes, highlighting a stress-driven neuroimmune axis that contributes to NPSLE-like phenotypes

Systemic lupus erythematosus (SLE) is a multisystem autoimmune disease that can involve the CNS and give rise to neuropsychiatric systemic lupus erythematosus (NPSLE) [44, 45]. NPSLE encompasses heterogeneous syndromes, including diffuse manifestations characterized by cognitive, mood, and behavioral abnormalities as well as focal neurological syndromes [45]. Because dNPSLE is biologically heterogeneous, no single experimental model fully recapitulates the human condition. Nevertheless, lupus-prone MRL/MpJ-Faslpr (MRL/lpr) mice provide a useful background in which disease-modifying factors can be examined [46, 47]. Within this framework, chronic psychological stress was examined as a potential modifier capable of unmasking dNPSLE-like phenotypes in genetically susceptible mice. In MRL/lpr mice, sustained stress conditions such as sleep disturbance induced marked behavioral alterations, including increased risk-taking behavior, reduced anxiety-like responses, heightened locomotor activity, and psychomotor agitation [19]. These changes were accompanied by region-specific neuroinflammatory responses, most notably microglial activation in the medial prefrontal cortex (mPFC), a brain region central to emotional regulation, executive function, and stress responsiveness [19, 48]. At the molecular level, stressed MRL/lpr mice exhibited elevated expression of IL-12/23 p40 in the mPFC and increased levels of this cytokine subunit in cerebrospinal fluid [19]. Parallel features were observed in patients with dNPSLE, who showed higher cerebrospinal fluid IL-12/23 p40 levels and greater mPFC atrophy than non-dNPSLE controls [19]. Moreover, pharmacological interventions targeting the IL-12/23 pathway, including receptor-directed blockade and TYK2 inhibition, ameliorated behavioral abnormalities and dendritic spine changes in stressed MRL/lpr mice [19]. Together, these results identify a stress-sensitive microglial IL-12/23 axis as a candidate mechanism contributing to dNPSLE-like pathology (Fig. 3). The present model does not yet fully resolve the activation state of the implicated microglia; for example, whether inflammatory, phagocytic, or partially homeostatic programs predominate over time. Likewise, the extent to which peripheral immune-cell infiltration into the mPFC contributes directly to the observed phenotype remains to be clarified. More broadly, NPSLE has also been linked to BBB dysfunction, immune-complex and complement-associated injury, type I interferon signaling, and brain-reactive autoantibodies; these pathways may intersect with stress-associated microglial activation rather than operate independently [49, 50]. From the perspective of earlier diagnosis, the stress-modified MRL/lpr model suggests that region-specific microglial activation and cerebrospinal fluid IL-12/23 p40 may serve as candidate markers of preclinical neuroinflammation. At present, however, it remains uncertain whether these signals are sufficiently sensitive or specific to identify presymptomatic dNPSLE in humans or how well they distinguish dNPSLE from other neuroinflammatory or psychiatric conditions. Because the inflammatory changes are spatially restricted and the human syndrome is heterogeneous, biomarker strategies will likely require integration of molecular markers with imaging, behavioral assessments, and additional immune parameters rather than reliance on a single cytokine axis.

## High-sensitivity detection of micro-inflammation and trace disease-associated markers

### Fluorescent nanodiamonds with nitrogen-vacancy centers as candidate sensors for micro-inflammation

FNDs are diamond nanoparticles containing NV centers that serve as photostable and biocompatible platforms for quantum sensing in biological environments [51]. Because NV fluorescence can be modulated by spin manipulation and read out by optically detected magnetic resonance or relaxometry, FNDs can report local magnetic noise, temperature, and related physicochemical changes with high spatial resolution while suppressing optical background from complex samples [51]. Further, FNDs have strong photostability, biocompatibility, subcellular localization, and active background suppression. These features make FNDs attractive candidate probes for interrogating spatially restricted inflammatory events.

In the context of micro-inflammation, the most direct readouts currently supported by the literature are local physicochemical consequences of inflammatory activation rather than cytokines themselves. Recent studies have shown that NV-based FND sensing can quantify free-radical generation in endothelial cells under shear stress, detect radical changes in functionalized FND systems in HeLa cells, and distinguish free-radical loads in synovial fluid and synovial cells from arthritis patients [52–54]. In addition, nanodiamond-based thermometry has been used to detect localized temperature changes associated with cellular activity [55]. These examples support the feasibility of using FNDs to probe oxidative stress, radical production, and other microenvironmental signatures that may accompany micro-inflammation or neuroimmune inflammation. By contrast, soluble inflammatory mediators such as IL-6, chemokines, or extracellular nucleic acids are unlikely to be sensed directly in a label-free manner by the NV center itself. Their detection would instead require assay architectures in which antibodies, peptides, aptamers, or other recognition elements are coupled to the FND surface, and target binding is converted into a measurable optical or magnetic readout.

FNDs are therefore best positioned as a candidate platform for detecting spatially restricted signatures of micro-inflammation through two complementary routes: direct sensing of local physicochemical changes, such as free-radical generation or nanoscale temperature variation, and engineered affinity-based assays for trace soluble biomarkers. However, there are still many challenges before FNDs are translated into earlier detection of chronic inflammatory or neuroimmune disease, including sensor delivery to disease-relevant sites, calibration in heterogeneous biological environments, the need for robust surface functionalization and transduction chemistry for soluble targets, and the lack of established evidence that FND-based assays can detect presymptomatic micro-inflammatory signals in human blood or cerebrospinal fluid.

### AI-assisted nanopore sensing as a candidate platform for trace biomarker detection

AI-assisted nanopore technology records ionic current waveforms generated when individual molecules or particles translocate through a nanoscale pore and then applies computational analysis to classify event patterns at the single-particle level. Its major strengths include single-particle analysis, rapid electrical readout, and compatibility with machine-learning-based classification. Recent studies have shown that coupling nanopore readouts to machine-learning models enables accurate discrimination of SARS-CoV-2 particles and variants in experimental and clinical settings [56, 57]. These studies are proofs-of-principle that complex ionic-current signatures can be classified with high accuracy in heterogeneous biological samples.

For chronic inflammatory and neuroimmune diseases, the most plausible nanopore targets are not disease concepts such as micro-inflammation per se, but specific analyte classes that may reflect micro-inflammatory states. Classes include extracellular nucleic acids such as microRNAs or cell-free nucleic-acid fragments, protein or peptide biomarkers analyzed through affinity-assisted carrier designs, and larger particulate targets if they generate sufficiently distinctive translocation signatures. Nanopore studies have already demonstrated aptamer-assisted protein detection, multiplex protein screening in human serum using DNA carriers, and femtomolar microRNA detection using biological nanopores [58–60]. These examples indicate that nanopore sensing canbe adapted to disease-relevant biomarkers beyond viruses.

Accordingly, if AI-assisted nanopores are to be linked to micro-inflammation, the most realistic near-term application would be the analysis of trace extracellular nucleic acids or engineered assays for selected soluble biomarkers, rather than direct label-free detection of cytokines and chemokines in unprocessed samples. These measurements would likely require affinity reagents, carrier molecules, or signal-amplifying workflows to convert target binding into reproducible waveform features that can be distinguished from background molecules. In a similar manner, disease-associated microRNAs or other nucleic-acid fragments might be detected by combining nanopore readout with amplification or DNA-computing strategies. However, before such technology is translated into earlier detection, there is still a need to carefully curate training datasets, establish the sensitivity of waveform features to sample preparation and buffer conditions, reliably identify clinically informative inflammatory analytes, and obtain evidence that the platform can detect presymptomatic micro-inflammatory biomarkers in human blood or cerebrospinal fluid.

## Future perspectives

The studies discussed in this perspective support a working model in which localized and low-abundance inflammatory programs may emerge before overt tissue damage or clear clinical manifestations. Within this framework, the IL-6 amplifier provides one mechanistic explanation for how non-immune cells can convert subtle perturbations into self-reinforcing inflammatory niches, whereas gateway reflex studies illustrate how neural activity can spatially organize such niches at defined vascular sites. Stress-associated dNPSLE-related mechanisms extend this view to region-specific brain inflammation and suggest that at least some neuropsychiatric manifestations may be preceded by localized neuroimmune remodeling [7–9, 14–19].

At the same time, these mechanisms should not be interpreted as exhaustive explanations of chronic inflammatory disease. Most direct evidence for gateway reflex-associated vascular micro-inflammation remains preclinical, dNPSLE is biologically heterogeneous, and the accessibility of equivalent presymptomatic signatures in human blood or cerebrospinal fluid remains uncertain [14–19, 45]. A major translational challenge is therefore not simply to increase analytical sensitivity, but to determine which early inflammatory changes are biologically informative, reproducibly measurable, and clinically distinguishable from infection, systemic inflammation, or other neurological and psychiatric conditions.

From a measurement perspective, FNDs and AI-assisted nanopores offer complementary solutions. FND-based approaches are currently best suited to detecting local physicochemical consequences of inflammation, such as radical generation or nanoscale temperature variation, whereas nanopore-based approaches may be especially useful for analyzing extracellular nucleic acids or engineered affinity-based signals derived from low-abundance soluble biomarkers [51–60]. Both platforms, however, should presently be regarded as candidate technologies rather than established diagnostic systems for presymptomatic chronic inflammatory disease.

Future progress will likely depend on integrating mechanistic immunology, quantitative sensing, and computational analysis. Important next steps include validation of candidate biomarkers in longitudinal human studies, development of surrogate readouts for spatially restricted inflammatory niches, standardization of sample handling and sensor calibration, and clarification of how candidate signatures relate to disease stage, tissue specificity, and treatment response. If these challenges are addressed, quantum- and nanoscale sensing may contribute to earlier and more mechanism-informed diagnosis of chronic inflammatory and neuroimmune disease.

## Data Availability

No datasets were generated or analysed during the current study.
